# Determinants of comprehensive knowledge of HIV transmission and prevention among women of reproductive age 15–49 years in Nigeria

**DOI:** 10.1371/journal.pgph.0003450

**Published:** 2024-07-30

**Authors:** Charles Nzelu, Uche Nzelu, Amara Uche Ugwunze, Ngozi Azodoh

**Affiliations:** 1 Department of Special Projects, Federal Ministry of Health Nigeria, Abuja, Nigeria; 2 National Examinations Council, Nigeria; 3 Department of Health Planning, Research and Statistics, Federal Ministry of Health Nigeria, Abuja, Nigeria; Aga Khan University, PAKISTAN

## Abstract

Comprehensive knowledge of HIV transmission and prevention has been reported as a necessary factor for ending the HIV epidemic. Women of reproductive age identified as a vulnerable group to HIV infection require this knowledge to prevent contracting HIV infection. Therefore, this study aimed to identify those factors that impact these women’s comprehensive knowledge of HIV transmission and prevention. This study used secondary data from the 2018 Nigerian Demographic and Health Survey. A weighted sample of women of reproductive age with complete data on the determinants and comprehensive knowledge of HIV was included in each analysis. The Chi-square test of Independence was used to determine the association between the determinants and the dependent variable. Bivariable and multivariable logistic regression analysis was done to predict the effects of the determinants on the comprehensive knowledge of women of reproductive age. Variables with a p-value of ≤ .01 were considered statistically significant determinants of comprehensive knowledge of HIV transmission and prevention. The findings showed that women of reproductive age with no or lower level of education, living in rural areas, poor wealth index, do not listen or listen less frequently to radio, and watch television less frequently were more likely to have comprehensive knowledge of HIV transmission and prevention at Alpha = .01. The number of women with comprehensive knowledge of HIV was low compared with those with no comprehensive knowledge. This is a concern that needs to be addressed. Further studies using primary data to validate the findings of this study that individuals with no or lower level of education, living in rural areas, poorer or middle wealth index, do not listen or listen less frequently to radio and do not watch or watch less frequently television were more likely to have comprehensive knowledge of HIV transmission and prevention are recommended.

## Introduction

At the end of 2020, about 37.7 million individuals are living with HIV globally [1]. New infections were 1.5 million, while 680,000 persons died from HIV/AIDS-related illnesses [1]. In Nigeria, the prevalence of HIV is 1.3% (male and female 15–49 years), with about 1.7 million individuals living with the virus at the end of 2020, and the country is rated fourth globally in terms of the HIV epidemic burden [1]. Women of reproductive age 15–49 years accounted for 56.47% of this number, with a prevalence rate of 1.6% in this age group [1]. Restricted educational and employment opportunities, poor negotiation of safe sex, poor economic empowerment, socio-cultural practices, and early or forced marriage increase the likelihood of HIV infection in women of reproductive age [2,3]. According to the 2018 Nigeria HIV/AIDS Indicator and Impact Survey, new infections are two times more likely to occur among women of reproductive age than men in the same age group [4]. Children become orphans and vulnerable due to the impact of HIV on this population group, resulting in adverse effects on the social and health systems in Nigeria [1].

In Nigeria, the 2013 Nigeria Demographic and Health Survey (NDHS) reported that 27% of women of reproductive age have comprehensive knowledge of HIV transmission and prevention, while the figure was reported as 46% in the 2018 NDHS [5,6]. This represents an increase of 19%. The 2018 NDHS reported that comprehensive knowledge of HIV prevention and control among young women aged 15–24 was 43%. It also reported that 71% of women of reproductive age in Nigeria knew that the use of condoms consistently and having sexual intercourse with only one uninfected partner reduces the risk of contracting HIV [6]. The 2018 NDHS definition of comprehensive knowledge of HIV transmission and prevention, which is the same as United Nations Children Fund (UNICEF) HIV comprehensive knowledge criteria, includes that an individual should know that consistent use of condoms during sexual intercourse and having just one uninfected faithful partner can reduce the chances of getting HIV, a healthy-looking person can have HIV, and rejecting the two most common local misconceptions about the transmission and prevention of HIV[6]. The definition or criteria entails that an individual knows the modes of HIV transmission and prevention and behaviours that minimize the spread of the virus, which are key to controlling the HIV epidemic. These modes and behaviours are necessary for individuals to engage in preventive health behaviours and reduce stigmatization and discriminatory practices against persons living with HIV/AIDS [7–9]. This study adapted the same criteria above for grading comprehensive knowledge, which includes knowing the two major ways of preventing the sexual transmission of HIV (using condoms and limiting sex to one faithful, uninfected partner), rejecting the two most common local misconceptions about HIV transmission and that a healthy-looking person can be HIV-positive [10]. However, we used the three most common local misconceptions about HIV transmission instead of the two used by UNICEF in the grading of comprehensive knowledge of HIV transmission and prevention.

Studies have reported that wealth index, level of education, access to radio, HIV testing, gender, place of residence, knowing of a place to test for HIV, use of traditional contraceptive methods, watching television, ownership of mobile phone, female head of household, marital status, ever tested for HIV, religion, age, age at sexual debut impacted on an individual’s comprehensive knowledge of HIV [11–20]. This study assessed the effects of some of these determinants of comprehensive knowledge of HIV transmission and prevention, namely educational level, wealth index, watching television and listening to the radio, marital status, age categories, place of residence, and religion that data were collected on in the 2018 NDHS it so that the findings will aid the development of informed policies, appropriate strategies, and interventions to tackle the HIV epidemic in Nigeria.

## Materials and methods

### Data source

This study used the 2018 NDHS data of women of reproductive age 15–49 years. This is a nationally representative study that used a two-stage stratified cluster design to select the study participants in Nigeria and collect data on the variables of interest using the women’s questionnaire. The detailed 2018 survey methodology has been described elsewhere [6].

### Variables measures

The measure for the dependent variable, comprehensive knowledge of HIV prevention and transmission, was created using the respondents’ responses to the UNICEF definition of HIV comprehensive knowledge for young people 15–25 years [10]. This definition was adapted to women of reproductive age 15–49 years and includes that they identify a) two major correct ways of HIV sexual transmission prevention, i.e., having sex with only one faithful uninfected partner and consistent use of condom during sexual intercourse; b) rejection of three most common local misconceptions about transmission of HIV (i.e., a) a person can get HIV from Mosquito bite, b) a person can get HIV by sharing food with an infected person, c) a person can get HIV by witchcraft or supernatural means; d) knowing that a healthy-looking person can have HIV infection. These were graded as < 6 = no comprehensive knowledge of HIV prevention and transmission if the women answered less than six questions correctly and coded as 0; 6 = comprehensive knowledge of HIV if the women answered all six questions correctly. The "I don’t know category" was treated as user-defined missing data. Sociodemographic variables identified in previous studies as determinants of comprehensive knowledge of HIV and which data were collected in the primary study were included in the study as predictor variables. The identified variables include Age Categories, Marital Status, Religion, Place of Residence, Wealth Index, Frequency of Listening to Radio, and Frequency of Watching Television. They were measured on the nominal scale except for Age Categories and Educational level, which were measured on the ordinal scale. The age of the women was categorized in ascending order into five-year interval groups. Place of Residence was coded as (Rural = 0; Urban = 1); Educational Level was recoded as (No Education = 0, Primary Education = 1, Secondary and Higher Education = 2); Religion recoded as (Christian = 1 (combining Catholic and Other Christian), Islam = 2); Marital Status categories recoded as Currently Married (combining married and living with a partner as if married) = 1, Not currently Married (combining other options) = 0; Household wealth index coded as (poor household wealth index = 0 (combining poorest and poorer), average household wealth index coded = 1, rich household wealth index = 2 (combining richer and richest); Frequency of Listening to Radio coded as (Not at all = 0, less than once a week = 1, at least once a week = 2); Frequency of Watching Television coded as (Not at all = 0, less than once a week = 1, at least once a week = 2.

### Data management and analysis

Statistical Package for Social Sciences (SPSS) Version 28 was used for the study analysis. The level of significance was set at Alpha = .05. Statistical analysis included women of reproductive age 15–49 years with complete data on variables of interest. Logistic Regression and Chi-Square tests of association were used to assess the relationship between the determinants (predictor variables) and comprehensive knowledge of HIV transmission and knowledge. Type 1 Error was controlled for due to the large sample size of the Demographic and Health Survey by reducing the level of significance from Alpha = .05 to .01[21]. Sample weights provided by the Demographic and Health Survey programs (DHS) were used for the analysis of the data to ensure the representativeness of the survey findings due to the non-proportional allocation of samples to states and places of residence, including the different response rates in the primary study [6]. Bivariate and multivariable regression analyses were conducted. Predictor variables that were not statistically significant at Alpha = .01 in the bivariate analysis were omitted from the multivariable regression analysis. Crude and adjusted odds ratios with a 99% confidence interval were reported for different categories of the predictor variables relative to their reference categories. Determinants with a *p-*value of ≤0.01 in the multivariable regression analysis were taken as a statistically significant factor associated with comprehensive knowledge of HIV transmission and prevention by women of reproductive age in Nigeria.

Because this study is a secondary data analysis of the 2018 NDHS, all women of reproductive age with complete data were included in the study analysis. The increase in sample size increased the precision of the study, reduced standard error, and increased statistical power [22]. The descriptive analysis of the variables of interest showed that none of the cases had more than 10% missing data. Therefore, listwise deletion was applied to handle the missing data [23]. Cross-tabulations were done between the predictors and dependent variables to get the valid weighted frequencies and percentages.

## Results

The study sample size was 41821 women of reproductive age; however, only the number of cases or respondents with complete data on the dependent variable (comprehensive knowledge of HIV transmission and prevention) were included in each analysis. The participants’ demographic characteristics are presented in Table 1. The sociodemographic variables included in this study are Age Categories, Educational Level, Place of Residence, Current Marital Status, Religion, Wealth Index, and Frequency of Listening to Radio and Watching Television. Women of reproductive age 15–19 years were highest in the sample with 19.4%, while those between 45–49 years were the least with 8.9%. Women with secondary and higher education were highest in the sample, with 52.5%, followed by those with no education (33.1%) and primary education (14.4%). Most of the women lived in rural areas and most were married,(69.6%). Most of the respondents do not have comprehensive knowledge of HIV transmission and prevention (97.2%).

**Table 1:** The frequency distribution of social and demographic characteristics of the women of reproductive age 15–49 years in Nigeria.

Predictors	Weighted Frequency (n)	Weighted Percent (%)
**Educational Level**		
No Education	13030	33.1
Primary Education	5673	14.4
Secondary/Higher Education	20636	52.5
**Age Categories (Years)**		
15–19	7636	19.4
20–24	6485	16.5
25–29	6838	17.4
30–34	5906	15.0
35–39	5215	13.3
40–44	3761	9.6
45–49	3496	8.9
**Place of Residence**		
Urban	18,499	47.0
Rural	20,839	53.0
**Religion**		
Christian	18,599	47.5
Islam	20,556	52.5
**Marital Status**		
Not Currently Married	11,954	30.4
Currently Married	27,385	69.6
**Household Wealth Index**		
Poor Household Wealth Index	13,764	35.0
Average Household Wealth Index	7,721	19.6
Rich Household Wealth Index	17,853	45.4
**Frequency of Listening to Radio**		
Not at all	16,741	42.5
Less than once a week	10,526	26.8
At least once a week	12,073	30.7
**Frequency of Watching Television**		
Not at all	18, 240	46.4
Less than once a week	7,753	19.7
At least once a week	13,344	33.9
**Comprehensive knowledge of HIV Transmission and Prevention**		
No Comprehensive HIV Knowledge of Transmission and Prevention	38,231	97.2
Have Comprehensive HIV Knowledge of Transmission and Prevention	1,108	2.8

**Fig 1:**
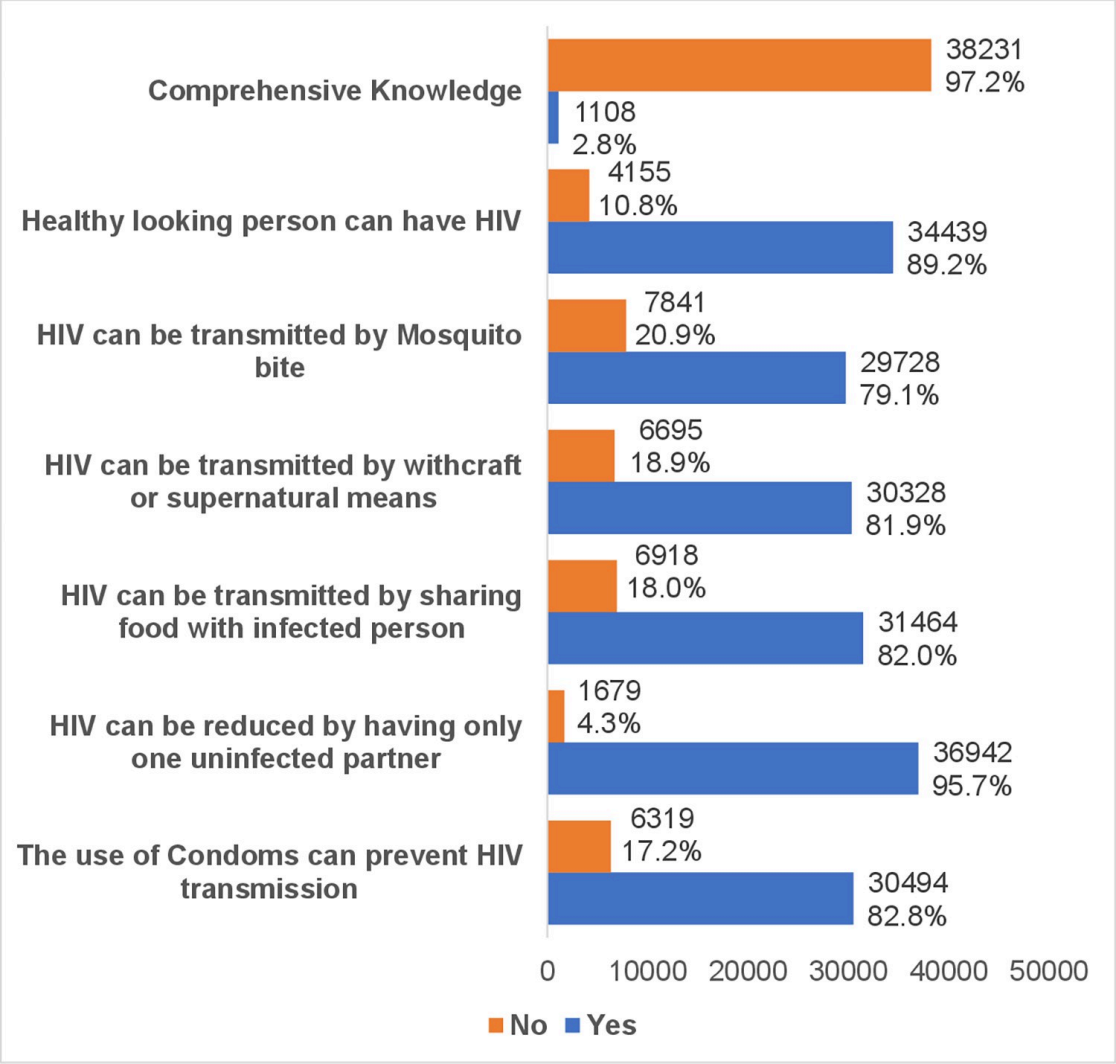
Frequency distribution of knowledge of HIV transmission and prevention among women of reproductive age 15–49 years in Nigeria.

The number of women with comprehensive knowledge was 1108 (2.8%), while those without comprehensive knowledge were 38231 (97.2%). Fig 1 shows the detailed frequency distribution of knowledge of HIV transmission and prevention among women of reproductive age 15–49 years in Nigeria.

Most of the women of reproductive age do not have comprehensive knowledge of HIV transmission and prevention.

Table 2 shows the Chi-square test of association between the women of reproductive age sociodemographic characteristic and their comprehensive knowledge of HIV transmission and prevention. The Chi-square result showed that educational level, place of residence, wealth index, and frequencies of listening to radio or watching television were statistically significantly associated with comprehensive knowledge of HIV transmission and prevention at Alpha **≤** 0.01. Age categories, current marital status, and religion were not statistically significantly associated with comprehensive knowledge of HIV.

**Table 2:** Chi-square test of association between comprehensive knowledge of HIV transmission and prevention of women of reproductive age (15–45 Years) in Nigeria and their sociodemographic characteristics.

Sociodemographic Variables	Yes (Have Comprehensive knowledge) n/%	No (No Comprehensive Knowledge) n/%	Chi-square (χ2), *p-*value
**Educational Level**			
No Education	537 (48.5)	12,492 (32.7)	
Primary Education	222 (20.0)	5,452 (14.3)	
Secondary Education or Higher Education	349 (31.5)	20,287(53.0)	χ2 = 202.21; (df = 2); *p-*value < .001
**Age Categories (Years)**			
15–19	234 (21.1)	7,402 (19.4)	
20–24	191 (17.2)	6,294 (16.5)	
25–29	185 (16.7)	6,653 (17.4)	
30–34	158 (14.3)	5,748 (15.0)	
35–39	128 (116)	5,087 (13.3)	
40–44	93 (8.4)	3,668 (9.6)	
45–49	119 (10.7)	3,377 (8.8)	χ2 = 11.37; (df = 6); *p-*value = .08
**Place of Residence**			
Rural	824 (74.4)	20,015 (52.4)	
Urban	284 (25.6)	18,215 (47.6)	χ2 = 209.48; (df = 1); *p-*value < .001
**Religion**			
Christianity	508 (45.9)	18091 (47.5)	
Islam	598 (54.1)	19,958 (52.5)	χ2 = 1.13; (df = 1); *p-*value = .29
**Marital Status**			
Not Currently Married	301 (27.2)	11,653 (30.5)	
Currently Married	807 (72.8)	26578 (69.5)	χ2 = 5.59; (df = 1); *p-value* = .02
**Household Wealth Index**			
Poor Household Wealth Index	617 (55.7)	13147 (34.4)	
Average Household Wealth Index	221 (19.9)	7500 (19.6)	
Rich Household Wealth Index	270 (24.4)	17583 (46.0)	χ2 = 250.59; (df = 2); *p-*value < .001
**Frequency of Listening to Radio**			
Not at all	626 (56.4)	16115 (42.2)	
Less than once a week	304 (27.4)	10222 (26.7)	
At least once a week	179 (16.2)	11894 (31.1)	χ2 = 130.64; (df = 2); *p-*value < .001
**Frequency of Watching Television**			
Not at all	708 (64.0)	17532 (45.9)	
Less than once a week	221 (20.0)	7532 (19.7)	
At least once a week	178 (16.1)	13166 (34.4)	χ2 = 182.93; (df = 2); *p-*value < .001

The crude and adjusted odds ratios from bivariate and multivariable logistic regression analysis are presented in Table 3. Women of reproductive age with No Education (AOR = 1.39: 99% CI; 1.11, 1.74) and those with Primary Education (AOR = 1.69: 99% CI; 1.33, 2.15) were statistically significantly more likely to have comprehensive knowledge of HIV transmission and prevention than those with secondary or higher levels of education. Women living in rural areas (AOR = 1.67: 99% CI; 1.35, 2.06) were statistically significantly more likely to have comprehensive knowledge of HIV transmission and prevention than those living in urban areas, while women with Poor Household Wealth Index (AOR = 1.55: 99% CI; 1.19, 2.03) were more likely to have comprehensive HIV knowledge than those with Rich Household Wealth Index. There was no statistically significant difference in the comprehensive knowledge of HIV between those with an Average Household Wealth Index (AOR = 1.27: 99% CI; .98, 1.65) and those with a Rich Household Wealth Index. Frequencies of listening to radio and watching television were statistically significantly associated with comprehensive knowledge of HIV. Those who do not listen to the radio at all (AOR = 1.69: 99% CI: 1.33, 2.15) and those who listen less than once a week (AOR = 1.67: 99% CI; 1.29, 2.16) were more statistically significantly more likely to have comprehensive knowledge of HIV than those who listen at least once a week. Age Categories, Current Marital Status, and Religion were not statistically significant in the bivariate regression model at Alpha = .01 and were removed from the multivariable regression analysis model.

**Table 3:** The bivariable and multivariable logistic regression analysis of determinants of comprehensive knowledge of HIV transmission and prevention of women of reproductive age.

Predictors	COR (99% C.I.), *p*-value	AOR (99% C.I.), *p*-value
**Educational Level**		p = .000
No Education	2.51 (2.09, 2.10)	1.39 (1.11, 1.74); *p* < 0.001
Primary Education	2.37 (1.89, 2.96)	1.69 (1.33, 2.15); *p* < 0.001
Secondary and Higher Education	*Ref*.	*Ref*.
**Age Categories (Years)**	.08	
15–19	.90 (.672, 1.21)	
20–24	.86 (.64, 1.17)	
25–29	.79 (.58, 1.07)	
30–34	.78 (.57, 1.07)	
35–39	.72 (.51,1.00)	
40–44	.72 (.50,1.03)	
45–49	Ref	*Ref*.
**Place of Residence**		p = .000
Rural	2.64 (2.21, 3.16)	1.67 (1.35, 2.06); *p <* .*001*
Urban	*Ref*.	*Ref*.
**Religion**		.000
Christianity	.94 (.80, 1.10), *p* = .29	
Islam	*Ref*.	
**Marital Status**		
Not Currently Married	.85 (.71, 1.02)	
Currently Married	*Ref*.	
**Household Wealth Index**		p = .000
Poor Household Wealth Index	3.05 (2.52, 3.69)	1.55 (1.19, 2.03); *p <* .001
Average Household Wealth Index	1.92 (1.52, 2.43)	1.27 (.98, 1.65); *p <* .019
Rich Household Wealth Index	*Ref*.	*Ref*.
**Frequency of Listening to Radio**		p = .000
Not at all	2.59 (2.07, 3.23); *p <* .001	1.69 (1.33, 2.15); *p <* .001
Less than once a week	.1.98 (1.55, 2.53); *p <* .001	1.67 (1.29, 2.16); *p* < .001
At least once a week	*Ref*.	*Ref*.
**Frequency of Watching Television**		p = .007
Not at all	2.98 (2.40, 3.71); *p <* .001	1.22 (.92, 1.62) *p* = .074
Less than once a week	2.17 (1.67, 2.82); *p-*value *<* .001	1.41 (1.07, 1.87); *p* = .002
At least once a week	*Ref*.	*Ref*.

## Discussion

This study found that comprehensive knowledge of HIV transmission and prevention was low (2.8%) among women of reproductive age in Nigeria. Both chi-square and logistic regression analysis results showed that educational level, place of residence, wealth index, and frequencies of listening to the radio and watching television were statistically significantly associated with the women of reproductive age 15–49 years in Nigeria’s comprehensive knowledge of HIV transmission and prevention at Alpha = .01. Age categories, religion of respondents, and marital status were not statistically significantly related to the women of reproductive age knowledge of HIV transmission and prevention in the bivariate model at Alpha = .01 and were omitted in the multivariable regression model. Interestingly, this study found that women with no education and primary education are more likely to have comprehensive knowledge of HIV transmission and prevention than women with higher education. This is surprising and a deviation from what most studies reported on this relationship. Studies have reported that women of reproductive age with higher levels of education are more likely to have comprehensive knowledge of education than those with lower levels of education [11,12,18,19,24]. However, a Nigerian study done in Akwa Ibom, Nigeria, reported that the level of education did not statistically significantly impact the comprehensive knowledge of HIV of young adolescents [25]. This was corroborated by another study done in Uganda [26]. This study found that women living in rural areas are more likely to have comprehensive knowledge of HIV than those in urban areas. This finding is surprising as rural dwellers are less likely to have access to electricity, radio, television, and internet, which will facilitate their access to information on HIV/AIDS [27]. Other studies done in Uganda, Nigeria, and Ethiopia [11,12,18,25,26] reported that women living in urban areas are more likely to have comprehensive knowledge than those living in rural areas. In this study, the household wealth index was significantly associated with comprehensive knowledge of HIV. Women in the poor household wealth index were statistically significantly more likely to have comprehensive knowledge of HIV than those in the rich household wealth index. This was supported by a study done in Ghana that reported a negative relationship between those in richer households and comprehensive knowledge of HIV [18]. Other studies [11,12,19,26] have reported that women of reproductive age in rich household wealth index are more likely to have knowledge of HIV prevention than those in poor household wealth index. This study finding is surprising because women from the rich household wealth index are supposed to be better educated and have more access to mass communication media (radio, television, internet, and telephone) from where they can get information about HIV transmission and prevention.

Non-exposure and less frequent exposure to media such as radio and television were statistically significantly associated with having comprehensive knowledge of HIV transmission and prevention in this study. Interestingly, women of reproductive age who did not listen to the radio and those who listened less than once a week were 1.69 and 1.67 statistically significantly more likely to have knowledge of HIV transmission and prevention than those who listened at least once a week, respectively. Also, women who watched television less than once a week were more likely to have comprehensive knowledge than those who watched at least once a week. This is not consistent with what other studies have reported. Other studies have reported that exposure to media such as radio and television facilitated more likelihood of having comprehensive knowledge of HIV transmission and prevention [12,20]. In contrast, some other studies reported no difference in comprehensive knowledge due to exposure and non-exposure to media [18,19,25]. This finding is surprising as radio and television stations in Nigeria provide information to Nigerians on HIV/AIDS either as part of corporate social responsibility or as paid advertisements by relevant stakeholders.

In Nigeria, NDHS provides the country with up-to-date basic demographic and health indicators estimates and information including the indicators relevant to achieving the Sustainable Development Goals. Therefore, the programme managers should be trained and involved in the conduct of NDHS so that their understanding of the processes and results will be useful in programme design, implementation, and evaluation in the communities and in developing strategies for improving Nigeria’s population health outcomes. Furthermore, the Demographic and Health Surveys conducted across many countries collect data on the required variables for defining HIV comprehensive knowledge, therefore, countries may not expend further funds in primary research to collect data on these needed variables.

A limitation of this study is the cross-sectional design of the primary research, which makes it unattainable to establish causality between the predictors and the dependent variable. Another limitation is recall bias, which may have impacted this study’s findings because the Demographic and Health Survey was a self-report of the respondents who participated in the survey.

## Conclusion

This study found that educational level, place of residence, household wealth index, and frequency of listening to radio and watching television statistically significantly impacted the knowledge of HIV transmission and prevention. The identification of these predictors of comprehensive knowledge of HIV will assist in designing informed policies, strategies, and interventions for the control of the HIV epidemic in Nigeria. Most of this study’s findings contradicted most studies on the relationship between the determinants and comprehensive knowledge of HIV transmission and prevention; therefore, further studies using primary data from the 2018 NDHS sample frame to validate this study’s findings are recommended.
